# Selenophosphate synthetase 1 (SPS1) is required for the development and selenium homeostasis of central nervous system in chicken (*Gallus gallus*)

**DOI:** 10.18632/oncotarget.16283

**Published:** 2017-03-16

**Authors:** Jin-Long Li, Wei Li, Xue-Tong Sun, Jun Xia, Xue-Nan Li, Jia Lin, Cong Zhang, Xiao-Chen Sun, Shi-Wen Xu

**Affiliations:** ^1^ College of Veterinary Medicine, Northeast Agricultural University, Harbin, 150030, P. R. China; ^2^ Heilongjiang Key Laboratory for Laboratory Animals and Comparative Medicine, Northeast Agricultural University, Harbin, 150030, P. R. China; ^3^ Key Laboratory of the Provincial Education Department of Heilongjiang for Common Animal Disease Prevention and Treatment, Northeast Agricultural University, Harbin, 150030, P. R. China

**Keywords:** selenophosphate synthetase 1, selenium homeostasis, central nervous system, development, chicken

## Abstract

Selenophosphate synthetase (SPS) is essential for selenoprotein biosynthesis. In two SPS paralogues, SPS1 was only cloned from a cDNA library prepared from avian organ. However, the biological function of SPS1 in chicken central nervous system (CNS) remains largely unclear. To investigate the role of avian SPS1 in the development and selenium (Se) homeostasis of CNS, fertile eggs, chicken embryos, embryo neurons and chicks were employed in this study. The response of SPS1 transcription to the development and Se levels of CNS tissues was analyzed using qRT-PCR. SPS1 gene exists extensively in the development of chicken CNS. The wide expression of avian SPS1 can be controlled by the Se content levels, which suggests that SPS1 is important in the regulation of Se homeostasis. The fundamental mechanism of these effects is that Se alters the half-life and stability of SPS1 mRNA. Therefore, SPS1 exerts an irreplaceable biological function in chicken CNS and Se homeostasis is closely related to the expression of SPS1. These results suggested that SPS1 was required for the development and Se homeostasis of CNS in chicken.

## INTRODUCTION

Selenium (Se) is a necessary micronutrient which is unique among trace elements in life activity. Se can reduce lipid peroxidation, elevate the activity of selenoenzyme and protect cells [1, 2], thus, it is important for the central nervous system (CNS). Se has been regarded as a component in bovine serum which is requisite to maintain neurons in serum-free media [3]. It has also been reported that Se acts a part in neurological disease [4]. The CNS is extremely sensitive to Se poisoning [1]. The neurotoxicity of Se compounds is demonstrated by its capability to induce motor neurons degeneration [5]. A recent study has confirmed that the brain competes for the utilization of Se under Se-compromised conditions, with concomitant effects on neurodegeneration and neurodevelopment [6]. Our previous study has reported that Se homeostasis exists in the brain, which means that Se content remains remarkably stable during Se supplementation in chicken [7]. The disruption of Se balance can induce neurodegeneration [8]. The alteration of selenoproteins expression is likely responsible for neuropathological alterations and behavioral changes in the brain with the excess and deficiency of Se [9].

Selenoproteins play an essential role in the protection of neurons [10] and the maintenance of brain function [11–14]. Se deficiency and selenoproteins expression reduction impair the development of brain including behavioral and functional defects [15–17]. Preferential retention of Se is largely connected with its transport to the brain and within it by selenoprotein [18]. Both neuronal and axonal degeneration as well as more moderate and potentially reversible neurite in the developing brain, are changed with the deletion of Selenoprotein P (Sepp1) [19]. It has become clear that Sepp1 is important to maintain brain Se and the neurons viability under the condition of Se-deficient [10]. Our previous study hypothesized that hierarchy of regulated the transcriptions of selenoproteome played a critical role of CNS Se metabolism and transport in birds [20].

Selenophosphate synthetase (SPS) was initially determined in bacteria and demonstrated to synthesize selenophosphate which is the active Se donor. ATP and SeH^−^, the metabolite of food or Se-containing substances *in vivo*, were catalyzed by SPS and transformed into selenophosphate (H_2_PO_3_SeH). H_2_PO_3_SeH reacted with PSer-tRNA^[Ser]Sec^ by Sec synthase (SecS) and formed Sec-tRNA^[Ser]Sec^ which led to the incorporation of Sec into the peptide chain of selenoproteins mRNA with SECp43, SECIS-binding protein 2 (SBP2) and selenocysteine-specific elongation factor (EFsec) [21]. Intriguingly, there are two SPS paralogues in higher eukaryotes called SPS1 and SPS2. It has been widely proposed that SPS2 synthesizes monoselenophosphate for generating Sec *in vitro* and *in vivo* studies. Nevertheless, SPS1, an enzyme which is highly homologous with SelD, is not involved in Sec synthesis in mammals [22], and a remarkable possibility is that a new pathway of Se utilization may be defined by SPS1 in animals [23]. SPS1 is also an essential mammalian enzyme which can control cell growth and is related with redox homoeostasis [24], and the enzyme it encodes lies on a selenium salvage system which recycles l-selenocysteine [25]. However, the exact biological function of the controversial enzyme, SPS1, has not been determined yet, especially in chicken.

Above all the aspects, the selenoproteins mRNA expression can be influenced by Se status in the nervous system. Of note, Roger et al. found that there was no SPS2 in avian [26]. Numerous clone studies of SPS2 have been reported but the SPS2 gene was not cloned in chickens. Whether SPS1 plays an essential role or not and the SPS1 mRNA expression regulated by Se in chicken CNS remain to be unclear. In consequence, this study aimed to investigate whether SPS1 was required for the development and Se homeostasis of CNS in chicken. Finally, we will provide new evidence regarding the unknown biological functionality of the SPS1 in birds.

## RESULTS

### Se content in CNS tissues

Se content in the chicken CNS tissues was shown in Supplementary Table 1. A dose-dependent increase of Se content was not shown in the cerebral cortex at 15d, cerebral nuclei at 35d and brain stem at 25d of the L-Se group compared with the C-Se group. Meanwhile, dose-dependent increases were shown in thalamus, cerebellum, medulla oblongata, marrow and sciatic nerve in L-Se group compared with C-Se group. When chickens fed diet was supplemented with 1.5 mg/kg Se (H-Se), Se levels did not change remarkably in the cerebral cortex, cerebral nuclei and marrow at 35d, thalamus, brain stem and medulla oblongata at 15d, and cerebellum at 15d and 25d compared with C-Se group, which indicated that Se homeostasis exhibited in chicken brain during Se supplementation, and the result was consistent with our previous study [7].

### Expression of SPS1 in the development of CNS tissues

**Figure 1:**
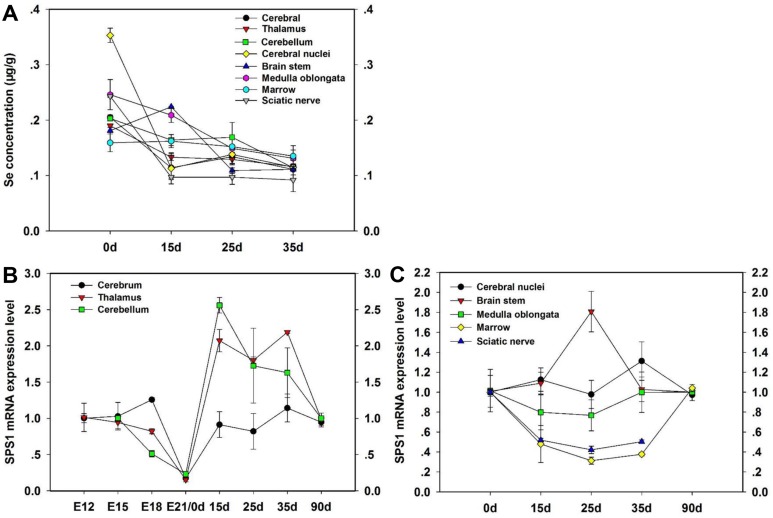
Expression of SPS1 in the development of CNS tissues Se content in the CNS tissues of chickens was determined at 0d, 15d, 25d and 35d (**A**). The SPS1 mRNA level in Cerebrum, Thalamus and Cerebellum was determined at E12, E15, E18, E21/0d, 15d, 25d, 35d and 90d (**B**). The SPS1 mRNA level in Cerebral nuclei, Brain stem, Medulla oblongata, Marrow and Sciatic nerve was determined at 0d, 15d, 25d, 35d and 90d (**C**).

To evaluate the expression of SPS1 in the development of chicken CNS, we measured the SPS1 mRNA level in the CNS tissues using qRT-PCR (Figure 1). The highest level of Se concentration was shown in cerebral nuclei at 0d, while the lowest level of Se concentration was shown at 35d in sciatic nerve. Almost Se concentration of all CNS tissues decreased at 15d, 25d and 35d compared with 0d (Figure 1A). SPS1 mRNA was the most abundant in cerebrum and least in cerebellum at E18. Then, the SPS1 mRNA level displayed a minimum level in cerebrum, thalamus and cerebellum at E21/0d, and increased significantly at 15d, 25d, 35d and 90d (Figure 1B). The SPS1 mRNA level in brain stem increased at 15d, however, further increases in time actually resulted in a reduction of SPS1 mRNA level after reaching a maximal level at 25d. In contrast, the SPS1 mRNA level in marrow decreased at 15d, reached the lowest level at 25d and then increased at 35d (Figure 1C). These results indicated that SPS1 expressed widely in chicken CNS tissues and the SPS1 mRNA level changed with the development of chicken CNS, which indicated that SPS1 might exert a necessary function in utilizing Se in chicken CNS.

### Effect of Se on the morphology and viability of embryo neurons

**Figure 2:**
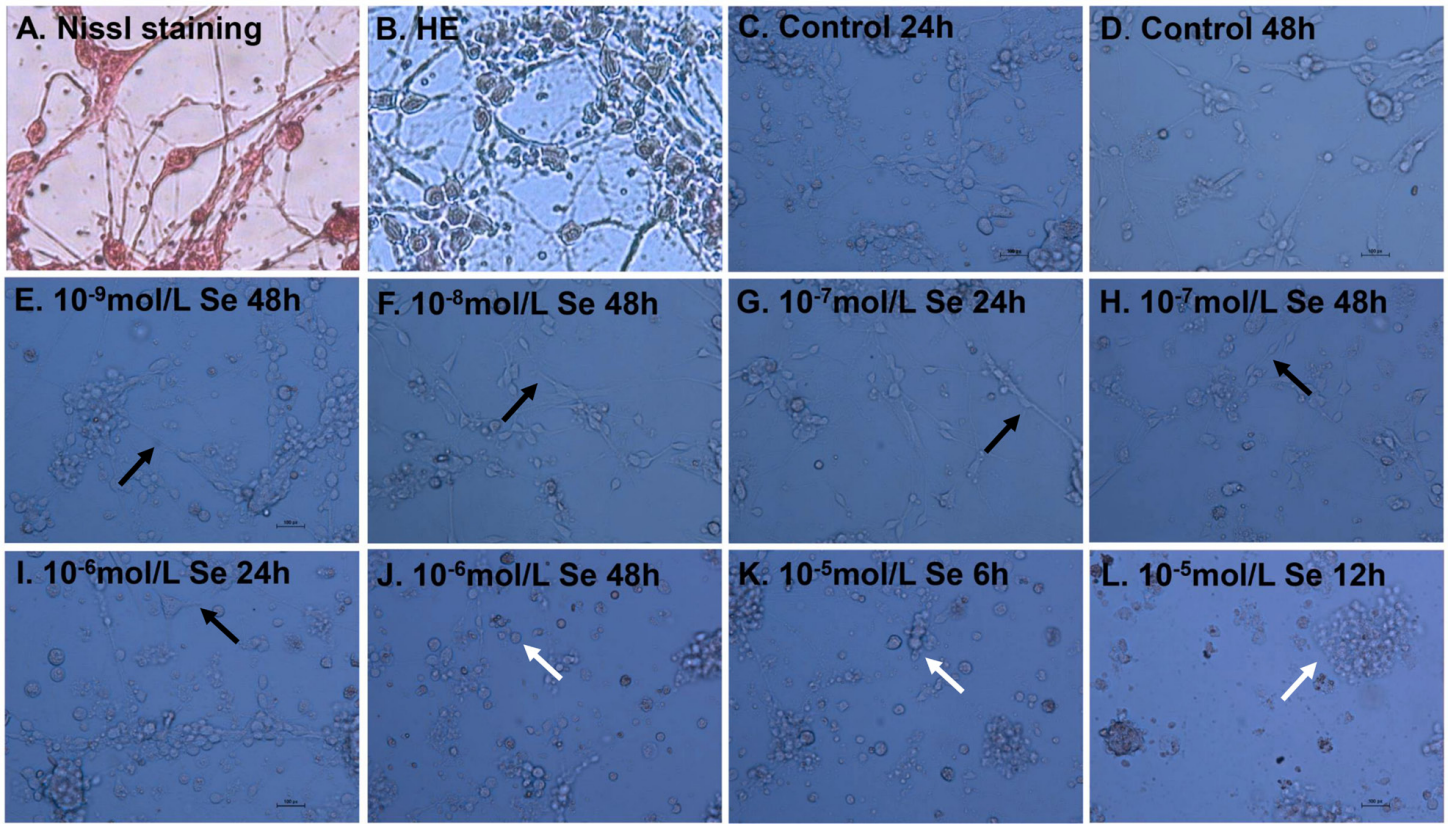
Effect of Se on the morphology and viability of embryo neurons Nissl staining (**A**) and Hematoxylin and eosin (HE) (**B**) of chicken embryo neurons. The chicken embryo neurons were treated with 0, 10^−9^, 10^−8^, 10^−7^, 10^−6^ or 10^−5^ mol/L of Se (sodium selenite) for 6, 12, 24 or 48 h (**C**–**L**). The morphology of treated and untreated neurons was visualized under the light microscopy (magnification: ×400, bar 50 μm). Note the neurite length and branches and the morphological alterations of neurons. The black arrows were used to indicate the neurite branches and length and the white arrows were used to indicate the shrinkage and fragmented neurites.

We have previously shown that Se treatment of chicken embryo neurons could cause the changes in the morphology and viability [9]. Hematoxylin and eosin (HE) and Nissl staining were used to confirm structural atrophy and showed the numbers of neurons (Figure 2A–2B). The chicken embryo neurons photomicrographs were shown in Figure 2C–2L. The morphological alterations (neurite length and branches) of neurons should be focused on significantly. The neurite branches and length of neurons in Se-I group (10^−9^ mol/L), Se-II group (10^−8^ mol/L) for 48 h and Se-III group (10^−7^ mol/L) for 24 h were more than those in the Control group (Figure 2D–2G). When compared with Control group, neurons showed a significant reduction in neurite length and branches when treated with 10^−7^ mol/L Se for 48 h and 10^−6^ mol/L Se for 24 h (Figure 2H–2I). Cell shrinkage and fragmented neurite were shown in Se-IV group (10^−6^ mol/L) for 48 h and Se-V group (10^−5^ mol/L) for 6 h and 12 h and it indicated that a higher dose Se (10^−6^ mol/L Se) resulted in cell death (Figure 2J–2L). These results suggested that chicken embryo neurons were sensitive to high Se concentrations and showed a dose-dependent and time-dependent decrease for the viability of neurons, which fell in line with our previous study [9].

### Effects of supplementary Se on SPS1 mRNA expression in embryo neurons

**Figure 3:**
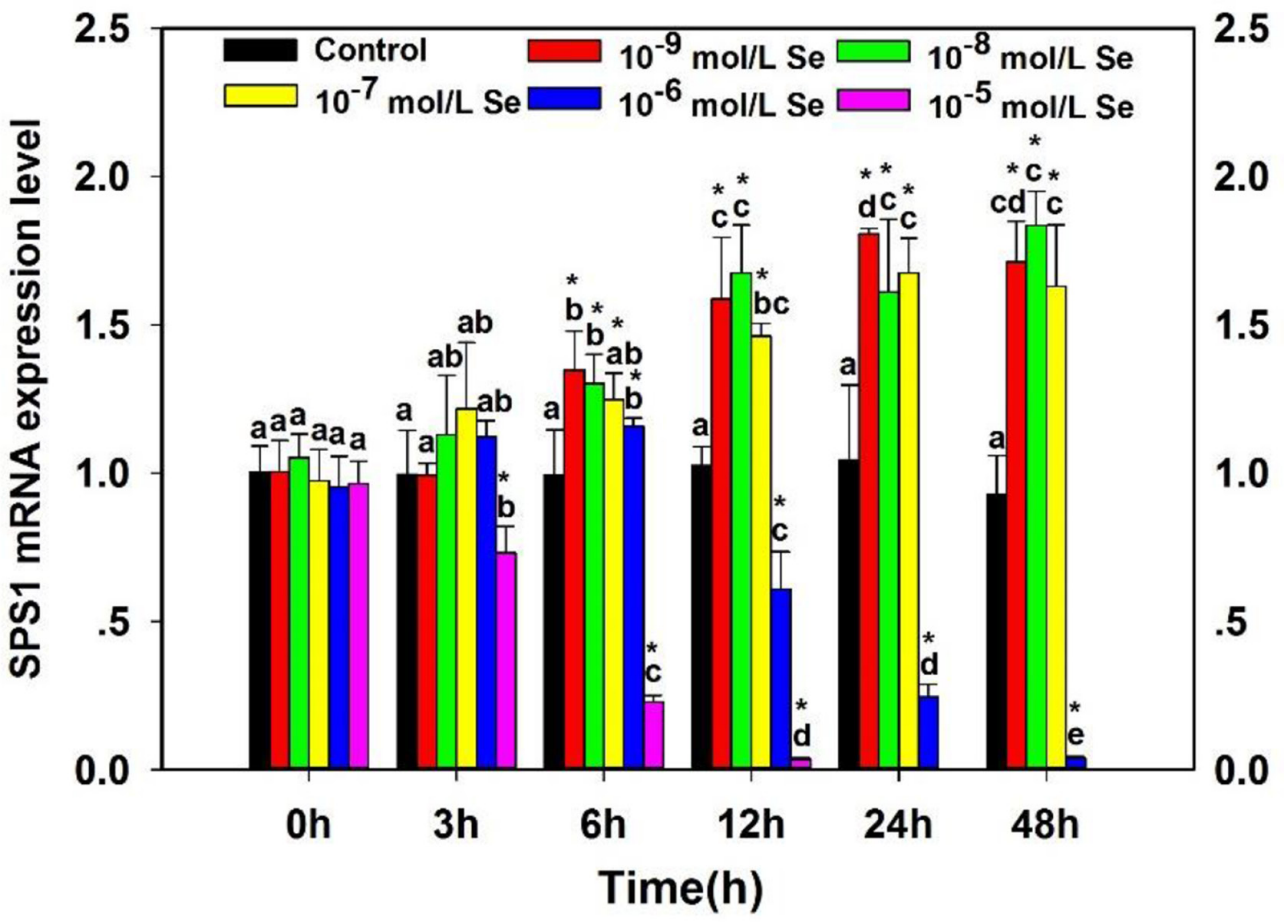
Effects of supplementary Se on SPS1 mRNA expression in embryo neurons The “*” is used to demonstrate significantly different from controls by one-way analysis of variance followed by a Tukey's multiple comparison test (*P < 0.05), and bars sharing a common letter (a or b or c or d or e) are not significantly different (*P* < 0.05).

To investigate the effect of supplementary Se on the SPS1 expression of neurons, the SPS1 mRNA level measured by qRT-PCR was shown in Figure 3. Treatment with Se upregulated the SPS1 mRNA expression in a time-dependent and does-dependent manner. The SPS1 mRNA expression decreased significantly in embryo neurons incubated with 10^−5^ mol/L Se at 3 h compared with the Control group (*P* < 0.05). The SPS1 mRNA levels showed maximal increases in the neurons incubated with 10^−9^ mol/L Se at 6 h and 24 h, 10^−8^ mol/L Se at 12 h and 48 h (*P* < 0.05). All of the SPS1 mRNA levels in neurons incubated with 10^−9^ mol/L, 10^−8^ mol/L and 10^−7^ mol/L Se were increased from 0–48 h, however, the SPS1 mRNA levels decreased significantly in neurons incubated with relatively high concentration (10^−6^ mol/L and 10^−5^ mol/L) of Se with the prolonged incubation time. The results indicated that the SPS1 mRNA level of chicken embryo neurons altered with the different Se concentration and the high Se concentration had a cytotoxicity for neurons.

### Effect of Se status on SPS1 mRNA stability

**Figure 4:**
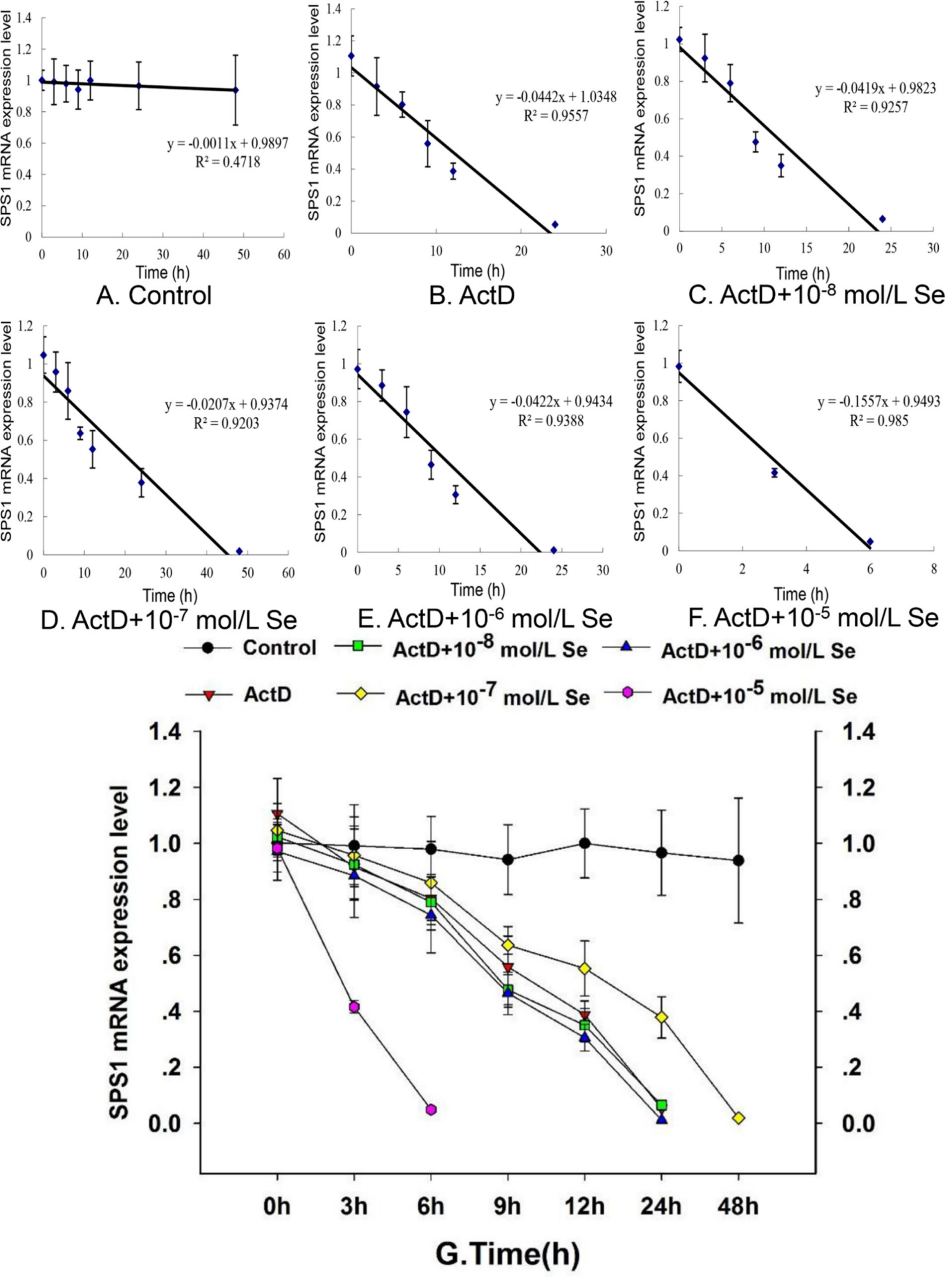
Effect of Se status on SPS1 mRNA stability The chicken embryo neuron monolayers were treated with PBS (Control) (**A**), 5 μg/mL ActD (ActD) (**B**), 5 μg/mL ActD and 10^−8^ mol/L Se (ActD+Se-I) (**C**), 5 μg/mL ActD and 10^−7^ mol/L Se (ActD+Se-II) (**D**), 5 μg/mL ActD and 10^−6^ mol/L Se (ActD+Se-III) (**E**) and 5 μg/mL ActD and 10^−5^ mol/L Se (ActD+Se-IV) (**F**) for 0 h, 3 h, 6 h, 9 h, 12 h, 24 h or 48 h. The mRNA stability was denoted with the SPS1 mRNA decay curve after 5 μg/mL ActD treatment mRNA level shown (**G**).

To investigate the mechanism between Se and SPS1, we examined the SPS1 mRNA half-life in chicken embryo neurons and the result was shown in Figure 4. A dose-dependent effect was displayed on the SPS1 mRNA half-life in neurons treated with Se. The SPS1 mRNA half-life was about 12.10 h when the embryo neurons were treated with ActD (Figure 4B), about 11.51 h when treated with ActD and 10^−8^ mol/L Se (Figure 4C), about 21.13 h when treated with ActD and 10^−7^ mol/L Se (Figure 4D), about 10.51 h when treated with ActD and 10^−6^ mol/L Se (Figure 4E) and about 2.89 h when treated with ActD and 10^−5^ mol/L Se (Figure 4F). It is worth noting that the SPS1 mRNA level in ActD+Se-II group (ActD and 10^−7^ mol/L Se) was higher than it in ActD group (ActD), however, a significant decrease of the SPS1 mRNA level was found in the neurons incubated with 10^−5^ mol/L Se (Figure 4G). The results here indicated that the neurons SPS1 mRNA stability incubated with 10^−7^ mol/L Se increased relatively and the neurons mRNA stability incubated with 10^−5^ mol/L Se reduced observably.

### Effect of Se status on SPS1 mRNA expression in embryo CNS

**Figure 5:**
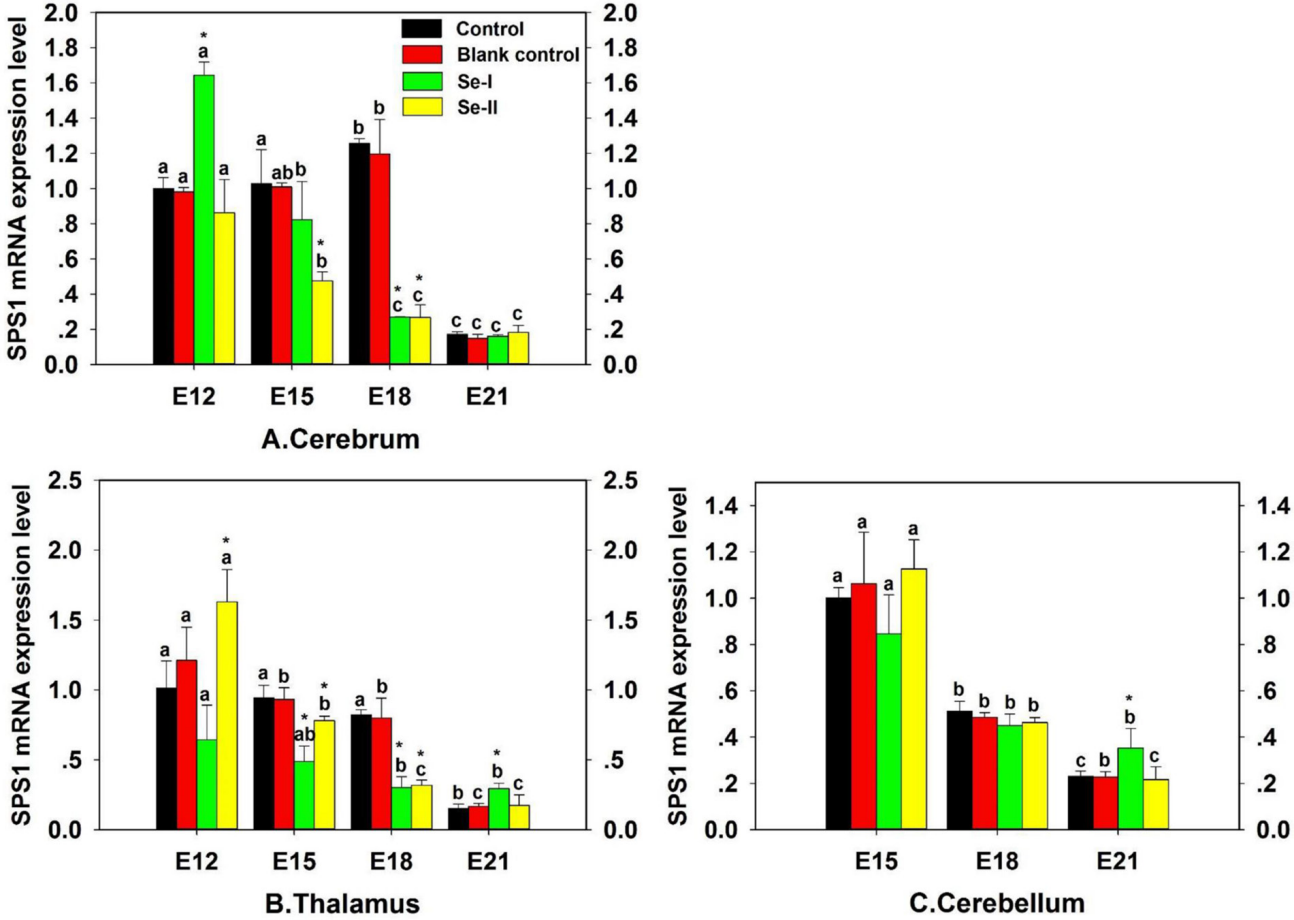
Effect of Se status on SPS1 mRNA expression in embryo CNS Cerebrum (**A**), Thalamus (**B**) and Cerebellum (**C**). The “*” is used to demonstrate significantly different from controls by one-way analysis of variance followed by a Tukey's multiple comparison test (*P < 0.05) and bars sharing a common letter (a or b or c or d or e) are not significantly different (*P* < 0.05).

To investigate the effect of Se status on SPS1 mRNA expression in chicken embryo CNS, the predetermined volume (0.1 μL/g egg) of PBS or Na_2_SeO_3_ solution was injected into the eggs to enhance the concentration of Se and the eggs were incubated, then the SPS1 mRNA level was detected and the result was shown in Figure 5. The SPS1 mRNA levels of embryo cerebrum and thalamus in Se-I group (inject Na_2_SeO_3_, 0.08 μg Se/mL in egg white) and Se-II group (inject Na_2_SeO_3_, 0.10 μg Se/mL in egg white) decreased significantly at E15 and E18, which indicated that excess Se could decrease the SPS1 mRNA level in embryo CNS. However, there was a significant increase in cerebrum in Se-I group and thalamus in Se-II group at E12 compared with Control group (*P* < 0.05) (Figure 5A–5B). Interestingly, the SPS1 mRNA levels were decreased in cerebrum, thalamus and cerebellum with the development of chicken embryo, of note, significant decreases were showed at E21 (*P* < 0.05), which indicated that the SPS1 mRNA displayed a minimum level in chicken embryo CNS at E21. There was no significant difference in Blank control group compared with Control group (*P* > 0.05), which indicated that the effect of injecting liquid into eggs before hatching on chicken embryo development was not observed.

### Effect of dietary Se status on SPS1 expression in chicken CNS

**Figure 6:**
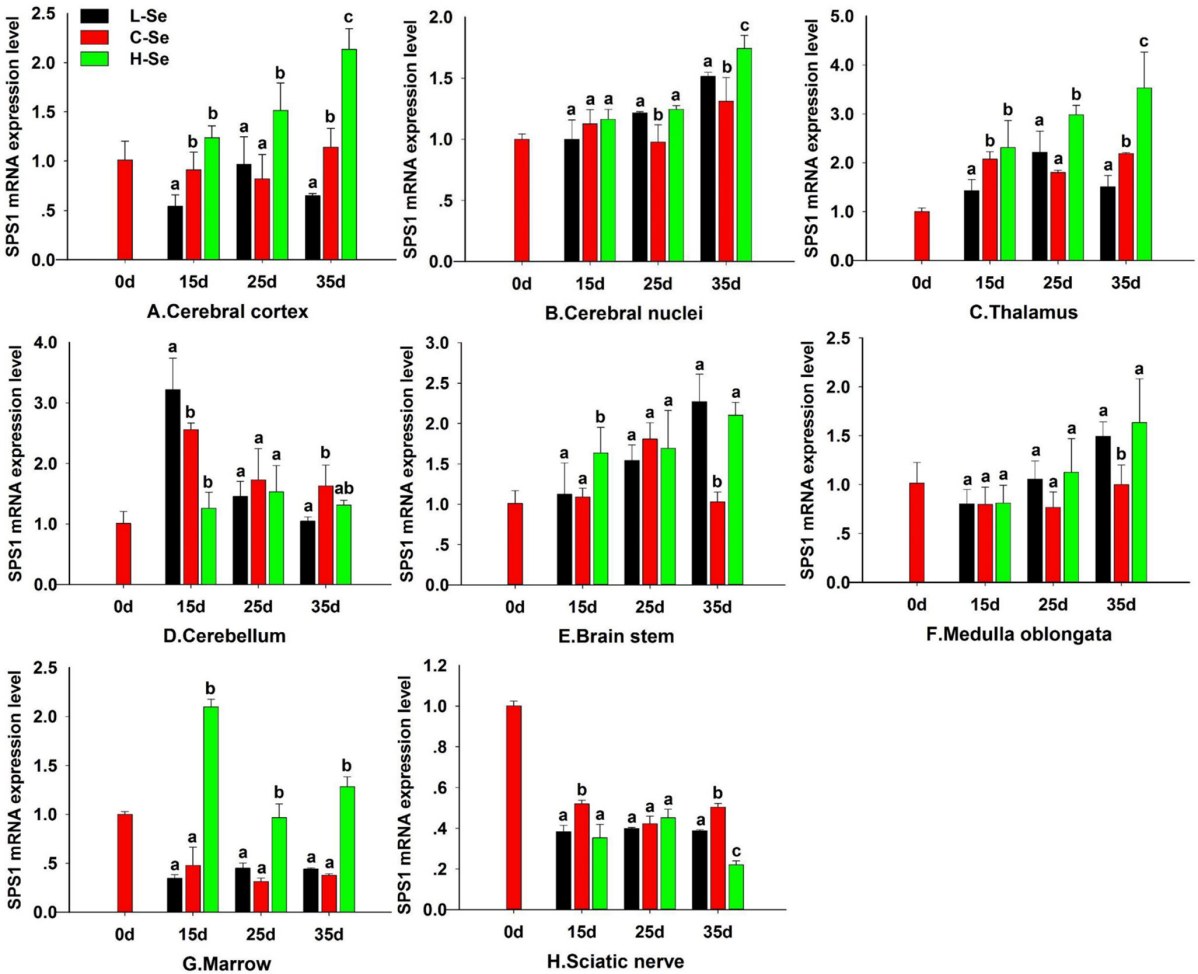
Effect of dietary Se status on SPS1 expression in chicken CNS Cerebral cortex (**A**), Cerebral nuclei (**B**), Thalamus (**C**), Cerebellum (**D**), Brain stem (**E**), Medulla oblongata (**F**), Marrow (**G**) and Sciatic nerve (**H**). L-Se group was fed with low Se diet which contained 0.033 mg/kg Se; C-Se group was fed with the diet containing 0.15 mg/kg Se; H-Se group was fed with the diet containing 1.5 mg/kg Se. Bars sharing a different letter are significantly different (*P* < 0.05).

To investigate the effect of dietary Se on the SPS1 expression in chicken CNS tissues, the chickens were fed with the diet containing Se and then the SPS1 mRNA level was determined and described in Figure 6. In our previous study, chickens which fed with the L-Se diet exhibited decreased motility and smaller body, the H-Se diet exhibited increased body weight [20]. The SPS1 mRNA levels were increased when chickens were fed with diets containing 0.033–1.5 mg/kg Se in cerebral cortex, thalamus and marrow at 15d, 25d and 35d, which indicated that there was a dose-dependent effect of Se status in cerebral cortex, thalamus and marrow (*P* < 0.05) (Figure 6A, 6C, 6G). Compared with C-Se group, the SPS1 mRNA levels in L-Se and H-Se group were increased in cerebral nuclei at 25d and 35d, brain stem and medulla oblongata at 35d (*P* < 0.05) (Figure 6B, 6E, 6F). There was a significant decrease in cerebellum at 15d (*P* < 0.05) (Figure 6D). A general decrease in the SPS1 mRNA level was observed in sciatic nerve at 15, 25d and 35d when compared with 0d (Figure 6H). Interestingly, the SPS1 mRNA level maintained stable in cerebellum, medulla oblongata, brain stem and sciatic nerve at 25d (*P* > 0.05). With the development of chick CNS, the SPS1 mRNA level increased in cerebral cortex, thalamus, cerebral nuclei, medulla oblongata and brain stem in H-Se group.

### Effect of supernutritional Se on SPS1 expression of chicken CNS

**Figure 7:**
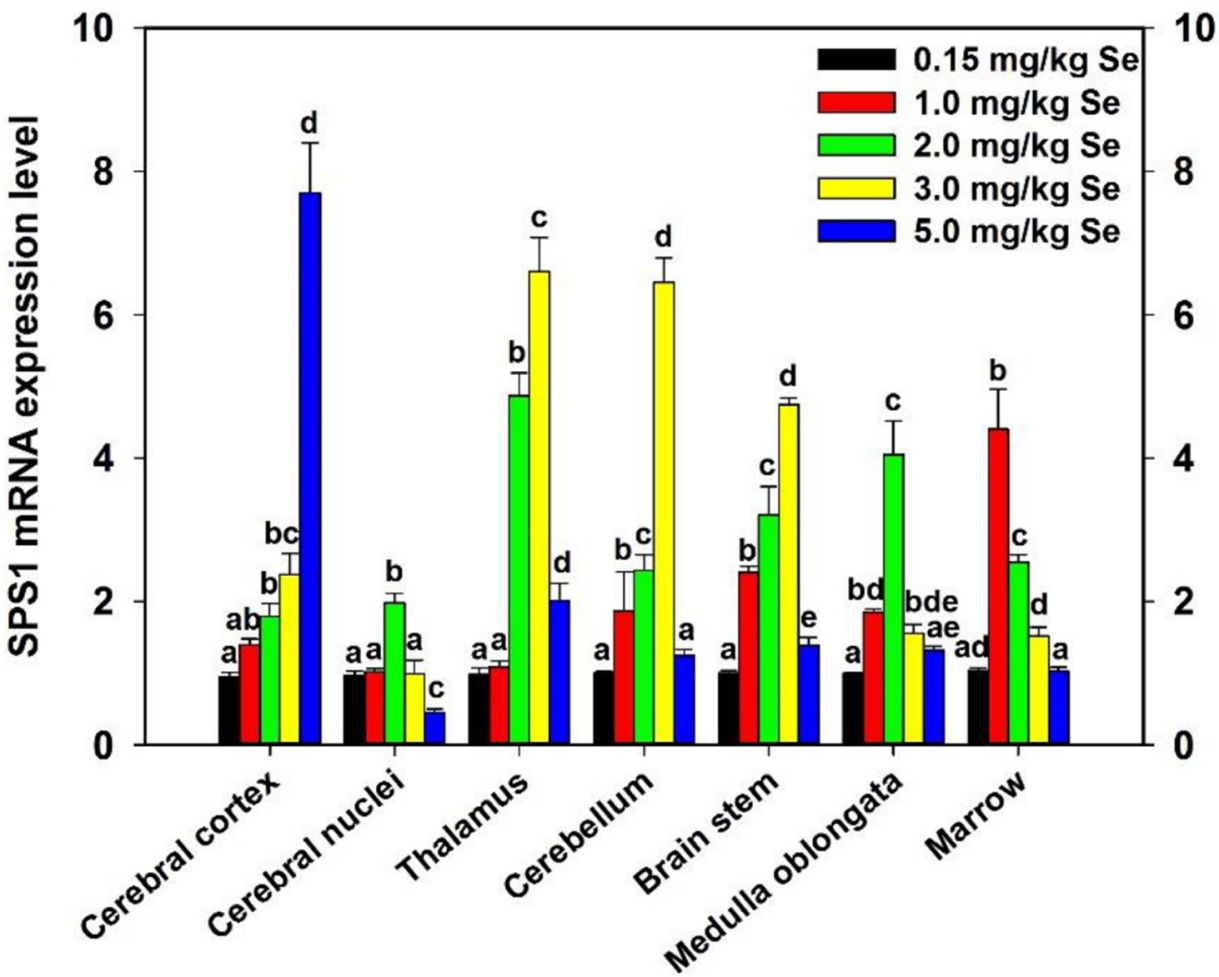
Effect of supernutritional Se on SPS1 expression of chicken CNS Control group was fed with the commercial diet which contained 0.15 mg/kg Se; Se-S- I group was fed with the Se-supplemented diet containing 1.0 mg/kg Se; Se-S- II group was fed with the Se-supplemented diet containing 2.0 mg/kg Se; Se-S- III group was fed with the Se-supplemented diet containing 3.0 mg/kg Se; Se-S- IV group was fed with the Se-supplemented diet containing 5.0 mg/kg Se. Bars sharing a different letter are significantly different (*P* < 0.05).

To determine the effect of supernutritional Se on the SPS1 mRNA expression of CNS, chickens were fed with the diet containing Se and then the SPS1 mRNA level was detected and the result was shown in Figure 7. The SPS1 mRNA levels were significantly increased in all Se-treated CNS tissues. And, of note, the maximum increases were found in cerebral nuclei and medulla oblongata of Se-S- II group, in thalamus, cerebellum and brain stem of Se-S- III group, and in marrow of Se-S- I group (*P* < 0.05). However, further increases of Se resulted in a decrease of the SPS1 mRNA abundance in the thalamus, cerebral nuclei, cerebellum, marrow, medulla oblongata and brain stem after reaching its maximal level. More remarkable, there was a Se upregulation in SPS1 mRNA expression in the cerebral cortex with 1.0–5.0 mg/kg dietary Se.

### Pearson correlation coefficient between tissues Se and SPS1 mRNA expression of chick CNS

Pearson correlation coefficient between tissues Se level and SPS1 mRNA expression of CNS tissues in chicks fed diets containing Se was showed in Supplementary Table 2. In chicken with low and high Se, the SPS1 mRNA level in cerebellum was negatively correlated with the tissues Se (*P* < 0.05) while the marrow showed a significant positive correlation (*P* < 0.01), but no significant correlation was found in other tissues (*P* > 0.05). In chicken with supernutritional Se, there were significant correlations between the SPS1 mRNA expression level and tissues Se in cerebellum (*P* < 0.01) and brain stem (*P* < 0.05).

## DISCUSSION

Se, exerting a number of beneficial effects on health through its pharmacotherapeutic efficacy against brain diseases [27], has been considered to be a necessary diet composition. The effect of Se on nerve has long been made an investigation into laboratory studies [28, 29]. Increasing evidences demonstrated an inhibitory effect displayed by Se against heavy metal neurotoxicity, such as lead, mercury, cadmium, and aluminum neurotoxicity [30, 31]. Se gives play to its biological functions basically through selenoproteins [32]. It has been reported that selenoproteins show a biological importance in the regulation of neuronal and metabolic homeostasis [33]. Our previous study found that supernutritional dietary Se did not significantly change the Se concentration in chicken brain [7]. In this study, Se homeostasis was observed in chicken CNS not only during Se supplementation but also Se deficiency. However, the mechanism needs further exploration.

SPS, the active selenium donor, was shown to synthesize selenophosphate. SPS2 is an isoform of SPS and when its mRNA was knocked out of the red beetle, silkworm and NIH3T3 cells, the selenoprotein biosynthesis would be lost, meanwhile, SPS2 was found to rescue the biosynthesis of selenoprotein which had been knocked out of NIH3T3 cells, while, SPS1 inhibition did not inhibit the synthesis of selenoprotein [22, 23]. However, SPS2 was not found in the avian tissues by Roger A. Sunde et al. [26]. SPS1, another isoform of SPS, lacks the capability to synthesize selenoproteins but may be involved in selenoprotein biosynthesis. [34]. It was reported that the knockout of SPS1 in *Drosophila* led to the lethality of embryonic and a remarkable increase in reactive oxygen species (ROS), which suggested that SPS1 participated in cell growth and the regulation of oxidative stress [35, 36]. Herein, we hypothesize that SPS1 may act an important part in chicken CNS. In this study, we identified that SPS1 was required for the development and Se homeostasis of CNS in chicken (*Gallus gallus*), and the SPS1 mRNA expression was regulated by Se status.

SPS1, aside from selenocysteine biosynthetic processes in human lung adenocarcinoma cells [25], and it has been reported to be involved in various physiological processes in conjunction with selenoproteins like imaginal disc development [37], cell proliferation [38], mitochondrion organization [39], glutaminemetabolism [40], stress responses [41], negative regulation of ROS homeostasis [42], and even neurogenesis [43]. In our study, SPS1 expressed widely in chicken CNS, and it was noted that SPS1 expression in CNS tissues was differ from other tissues. In addition, the SPS1 expression level was significantly decreased with a top Se concentration, and then increased with relative low Se concentration in CNS tissues, which suggested that SPS1 played a key role in utilizing Se in chicken CNS.

Se is beneficial to the development of embryo. It has been demonstrated that dietary organic Se can improve embryonic development of pig by enhancing the transference of maternal Se to 30d pig conceptuses in early pregnancy [44]. The recent research demonstrated that maternal Se could enhance antioxidative capacity and reduce oxidative damage and ROS concentration of chick embryo [45]. Of note, the biological functions of Se are largely effected by selenoproteins, and SPS1 exerts a potential function in the biosynthesis of SelW which is a kind of selenoprotein [46]. In this study, the expression of SPS1 was decreased significantly when the eggs were treated with Se. The results demonstrated that the SPS1 mRNA expression in chicken embryo CNS was significantly regulated by the content of Se injected into the eggs, and excess Se could decrease the SPS1 mRNA expression. In particular, differences in variation trend of the SPS1 mRNA expression were shown in different CNS tissues, which suggested that SPS1 was regulated tissue-specifically by Se.

Brain has high priority for Se uptake with relatively low nutritional Se [47]. In this regard, there must be a specific mechanism for the uptake and storage of Se, in which selenoprotein is considered to be a key factor for Se preferential homeostasis in CNS [48]. Our previous study indicated that Se homeostasis existed in cerebral cortex and thalamus particularly [7]. Herein we found that the SPS1 mRNA expressions were decreased significantly in chicken cerebral cortex and thalamus with low Se diet. The above results performed on the alteration of SPS1 mRNA expression demonstrated that the Se utilization of SPS1 was decreased with Se deficiency to maintain the stability of Se content in chicken CNS.

In our previous study, Se homeostasis of chicken brain tissues was preferentially maintained even with Se supplementation in dietary [7]. The selenoproteins expressions in certain tissues were differentially controlled by dietary Se levels [49, 50]. Nevertheless the brain with an insufficient Se intake shows a pronounced reduction of selenoprotein expression under these conditions [51]. This tissue-specific hierarchy for Se uptake or retention mechanism exists by which the brain maintains its required Se levels, even under suboptimal nutritional conditions [1]. In the present work, the expression of SPS1 was increased with the excess Se in chicken CNS tissues, which indicated that the SPS1 expression was controlled by Se level. Similarly, to maintain the stability of Se content in CNS, the Se utilization of SPS1 was increased with the supernutritional Se. In particular, the regulation was described significantly in cerebral cortex, which was reasonable to assume that SPS1 might be important in chicken cerebral cortex.

It has been determined that appropriate Se concentration is beneficial to the survival and growth of cells, nevertheless, higher Se concentration is prohibitive for the cells growth and leads to cells death [52]. Our previous study has demonstrated that Se can enhance the neurite outgrowth, whereas the high Se concentrations show neurons neurotoxicity in contrast. Meanwhile, we have found that Se can increase the levels of SelW mRNA and elevate SelW mRNA half-life in embryo neurons of chicken, while they decrease with high Se concentrations. The results described above demonstrated that SelW could protect developing neurons from oxidative attack of exogenous and endogenous origin [9]. In our study, the expression of SPS1 was determined in chick embryo neurons to investigate the biological function of SPS1 in neurons. The expression of SPS1 mRNA was sensitive to the content of Se, and it was decreased by relatively high concentrations of Se, and in contrast, appropriate Se was found to increase the SPS1 mRNA expression levels. And the effect of Se on SPS1 mRNA expression level showed a dose-dependent and time-dependent. The results indicated that Se might influence neurons through the alteration of the SPS1 expression in chick embryo neurons. Or maybe SPS1 involved in the biosynthesis of SelW, altering the viability of neurons. However, the hypothesis should be demonstrated in the further studies.

The mRNA half-life determination is helpful to understand gene expression and mechanisms of transcripts level in response to developmental cues and environmental changes. Se can alter the SepSecS and SelW mRNA stability in neurons [7, 9]. We also inquired into the mechanism that Se regulated the expression of SPS1 in chicken neurons. In our study, the SPS1 mRNA half-life was enhanced with appropriate Se concentration and reduced with the high Se concentration in contrast. The data above indicated that Se might alter the stability of SPS1 mRNA in neurons. The results indicated that the SPS1 mRNA post-transcriptional stabilization was an important mechanism for the elevation of Se-induced and decreased expression of SPS1. Meanwhile, the Pearson correlation coefficient between tissues Se concentration and the SPS1 mRNA abundance in the CNS tissues was analyzed. There was a remarkable direct relationship between Se content in tissues and the SPS1 mRNA abundance in the cerebellum and marrow of chickens with low and high Se and cerebellum and brain stem of chickens with supernutritional Se, which supported the findings.

In summary, SPS1 exists extensively in the development of chicken CNS. The wide expression of avian SPS1 can be controlled by the Se content levels, which suggests that SPS1 acts a part in the regulation of Se homeostasis. The fundamental mechanism of these effects is that Se alters the half-life and stability of SPS1 mRNA. Therefore, SPS1 exerts an irreplaceable biological function in chicken CNS and Se homeostasis is closely related to the expression of SPS1. These results suggested that SPS1 was required for the development and selenium homeostasis of CNS in chicken. The avian SPS1 biological function needs to be further studied using genetic modification models.

## MATERIALS AND METHODS

### Ethics statement

**Figure 8:**
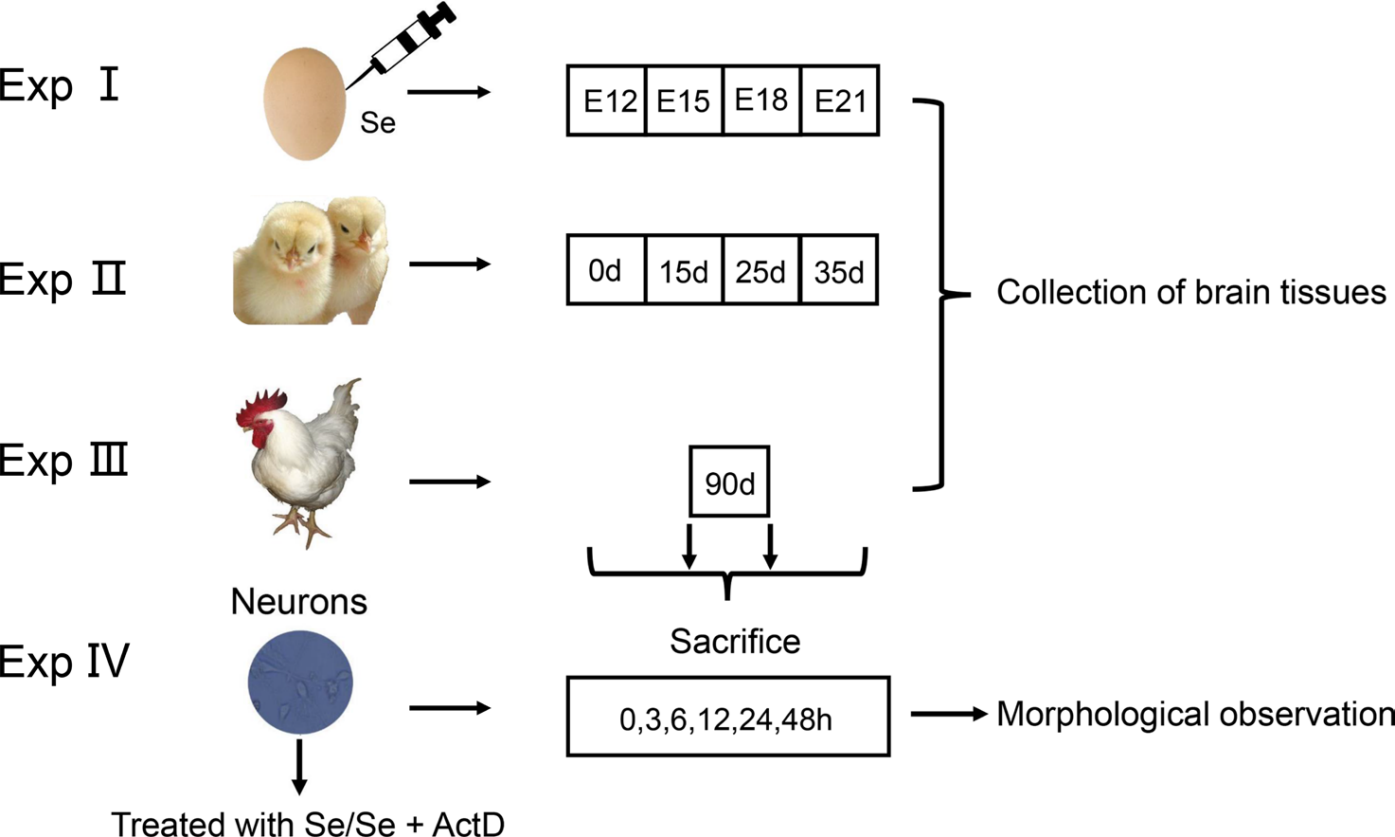
Experimental design

All experimental procedures were approved by the Institutional Animal Care and Use Committee of Northeast Agricultural University (NEAU). Methods were carried out in accordance with the ethical standard of the institution. The studies were divided into three sections: chicken embryos, *in vivo* and *in vitro* (Figure 8).

### Experiment in embryo

#### Exp I: chicken embryo

*Gallus domesticus* (HY-LINE VARIETY WHITE) fertile eggs were obtained from a commercial (Xian Feng Co. Ltd., Harbin, China). These eggs were cleaned, labeled and weighed 61.2 ± 2.0 g and the air cell was located by egg candle. A 1 mm in diameter hole was drilled into the center of the air cell. On the basis of the egg's weight, a microinjector was used to inject predetermined volume (0.1 μL/g egg) of PBS or Na_2_SeO_3_ (Sigma, USA) solution into the hole. The hole was sealed, and the eggs were left upright for 1 h at room temperature. Eggs were randomly divided into four groups as follows: Control (untreated, 0.07 μg Se/mL in egg white), Blank control (inject PBS, 0.07 μg Se/mL in egg white), Se-I group (inject Na_2_SeO_3_, 0.08 μg Se/mL in egg white), Se-II group (inject Na_2_SeO_3_, 0.10 μg Se/mL in egg white). These eggs were incubated in an automatic incubator. The cerebrum, thalamus and cerebellum of chicken embryos were gathered at 12-day-old (E12), 15-day-old (E15), 18-day-old (E18), and 21-day-old (E21) of hatching period. These tissues were frozen in liquid nitrogen and stored at −80°C until use.

### Experiment *in vivo*

#### Exp II: chickens fed with low and high Se diet

One hundred and eighty male chickens (1-day-old; XianFeng Co. Ltd., Harbin, China) were divided into three groups (60 chickens/group) at random. L-Se group was fed with low Se diet which contained 0.033 mg/kg Se (Longjiang County, Heilongjiang Province). C-Se group was fed with the diet containing 0.15 mg/kg Se with Na_2_SeO_3_ which increased the Se content of L-Se group. H-Se group was fed with the diet containing 1.5 mg/kg Se with Na_2_SeO_3_ which increased the Se content of L-Se group. Each group was fed regularly and independently for 35 days and gathered the CNS tissues (cerebral cortex, thalamus, cerebral nuclei, cerebellum, medulla oblongata, brain stem, marrow and sciatic nerve) at 0d, 15d, 25d and 35d respectively. These CNS tissues were frozen in liquid nitrogen and stored at −80°C until use.

#### Exp III: chickens fed with supernutritional Se diet

1-day-old male chicks (Xian Feng Co. Ltd., Harbin, China) were randomly divided into five groups (10 chickens/group) with Na_2_SeO_3_ for 90 days as follows:

Group 1(Control) was fed with the commercial diet which contained 0.15 mg/kg Se; Group 2 (Se-S- I) was fed with the Se-supplemented diet containing 1.0 mg/kg Se; Group 3 (Se-S- II) was fed with the Se-supplemented diet containing 2.0 mg/kg Se; Group 4 (Se-S- III) was fed with the Se-supplemented diet containing 3.0 mg/kg Se; Group 5 (Se-S- IV) was fed with the Se-supplemented diet containing 5.0 mg/kg Se. Chickens were fasted overnight. The CNS tissues (cerebral cortex, thalamus, cerebral nuclei, cerebellum, medulla oblongata, brain stem and marrow) were quickly removed, and frozen instantly in liquid nitrogen and then stored at −80°C pending analysis.

### Experiment *in vitro*

#### Exp IV: Chicken embryo neuron

##### Preparation of chick embryo neurons

Primary cultured neurons were prepared in accordance with a modified protocol [53]. Chick embryo cerebral hemispheres were dissected on day 8 and cultivated in 6-well poly-D-lysine-coated cell culture plates (Sigma, USA). Blood and adhering meningeal membranes were cleaned aseptically. Cells were cultured by enzymes digesting method and filtered. The dispersed cells were plated in DMEM which contained HEPES, supplemented with antibiotics, 2 mM glutamine and 10% fetal bovine calf serum.

### Neuron cultures and treatments

2 mg/mL cytarabine (Sigma, USA) was supplemented into the culture medium after 24h. Then, the cell monolayers were washed three times with PBS (0.1 M, pH 7.4) and incubated in neurobasal culture medium at 37°C with 5% CO_2_ humidified atmosphere. After being cultured for 48h, neurons were respectively cultured in 2 mL fresh complete medium and incubated in the presence of 0 (Control), 10^−9^ (Se-I), 10^−8^ (Se-II), 10^−7^ (Se-III), 10^−6^ (Se-IV) or 10^−5^ (Se-V) mol/L of Se as Na_2_SeO_3_ for 0 h, 3 h, 6 h, 12 h, 24 h and 48 h.

### Determination of the morphology of Neurons

HE and Nissl staining were used to learn the changes of the modality and number of chicken embryo neurons. The morphology of neurons was determined with a light microscopy (Eclipse-Ti, Nikon, Japan) at × 400 magnifications.

### Determination of Se concentration

Se content was determined according to Li et al. and Hasunuma et al. [7, 54]. The measurement is grounded on the following principle: the Se response in acid contained in samples acid digestion and converted to selenite.

### Determination of SPS1 mRNA level by qRT-PCR

Total RNA were extracted from the neuron monolayers and the tissue samples (50 mg tissue; *n* = 5/diet group) using TRIZOL (Invitrogen, China) following the instructions. 40 μL diethyl-pyrocarbonate-treated water was used to re-suspend the dried RNA. Oligo dT primers and TransScript Reverse Transcriptase were used to synthesize the first-stand cDNA (Beijing TransGen Biotech Co. Ltd., P.R. China) according to the instructions. The cDNA was diluted 10 times with sterile water and stored at −20°C before use.

Primers for the SPS1 genes (NM_001164084.1, Forward: 5′- CTGCTGGACTTATGCACAC -3′, Reverse: 5′- ACACCTCATTTCGCTGCT -3′, 108bp) and the β-actin (NM_205518.1, Forward: 5′- CCGCTCTATGAA GGCTACGC -3′

Reverse: 5′- CTCTCGGCTGTGGTGGTGAA -3′, 128 bp), Glyceraldehyde 3-phosphate dehydrogenase (GAPDH) (K01458, Forward: 5′-AGAACATCATCCC AGCGT-3′

Reverse: 5′-AGCCTTCACTACCCTCTTG-3′, 182 bp) were designed using Primer Analysis Software (Oligo 7.24, Molecular Biology Insights, Inc. USA). qRT-PCR was used to determine mRNA quantities using a LightCycler^®^ 480 Real Time PCR System (Roche Applied Science, CA, USA) and GoTaq^®^ qPCR Master Mix (A6001, Promega, USA). Only one peak of the melting curve was shown for each PCR product. All data was normalized to the house keeping gene, GAPDH and β-actin. Pfaffl method was used to calculate relative changes on mRNA expression [55].

### Determination of SPS1 mRNA half-life

The determination of SPS1 mRNA half-life was executed on the basis of the previous study [56]. The chicken embryo neurons were divided into six groups. The medium of neurons was treated with PBS (Control), 5 μg/mL actinomycin D (ActD, Sigma, USA) (ActD), 5 μg/mL ActD and 10^−8^ mol/L Se (ActD+Se-I), 5 μg/mL ActD and 10^−7^ mol/L Se (ActD+Se-II), 5 μg/mL ActD and 10^−6^ mol/L Se (ActD+Se-III) and 5 μg/mL ActD and 10^−5^ mol/L Se (ActD+Se-IV)for 0 h, 3 h, 6 h, 9 h, 12 h, 24 h or 48 h and the SPS1 mRNA levels were measured by qRT-PCR. ActD was used to stop the transcription of neurons. The half-life of the mRNA was deduced from the SPS1 mRNA attenuation curve as the time point after ActD treatment, in which 50% of the initial mRNA level remained.

### Pearson correlation coefficient between tissues Se content and SPS1 mRNA levels

The relationship between CNS tissues Se concentrations and the abundance of SPS1 mRNA were assessed by Pearson correlation coefficient. Differences were considered to be significant at *P* < 0.05 [57].

### Statistical analysis

Statistical analysis of the SPS1 expression in chicken CNS was analyzed using One way-analysis of variance. SigmaPlot 12.5 (SigmaPlot Software Inc., USA) was used to draw the Figures. Tukey's honestly significant difference test was used to assess the differences in the means of data for post hoc multiple comparisons. The results were expressed as mean ± S.D. of different groups. Differences were supposed to be significant at *P* < 0.05.

## SUPPLEMENTARY TABLES



## References

[1] Brauer AU, Savaskan NE (2004). Molecular actions of selenium in the brain: neuroprotective mechanisms of an essential trace element. Rev Neurosci.

[2] Savaskan NE, Bräuer AU, Kühbacher M, Eyüpoglu IY, Kyriakopoulos A, Ninnemann O, Behne D, Nitsch R (2003). Selenium deficiency increases susceptibility to glutamate-induced excitotoxicity. FASEB J.

[3] Yan J, Barrett JN (1998). Purification from bovine serum of a survival-promoting factor for cultured central neurons and its identification as selenoprotein-P. J Neurosci.

[4] Vinceti M, Maraldi T, Bergomi M, Malagoli C (2009). Risk of chronic low-dose selenium overexposure in humans: insights from epidemiology and biochemistry. Rev Environ Health.

[5] Maraldi T, Riccio M, Zambonin L, Vinceti M, De Pol A, Hakim G (2011). Low levels of selenium compounds are selectively toxic for a human neuron cell line through ROS/RNS increase and apoptotic process activation. Neurotoxicology.

[6] Pitts MW, Kremer PM, Hashimoto AC, Torres DJ, Byrns CN, Williams CS, Berry MJ (2015). Competition between the Brain and Testes under Selenium-Compromised Conditions: Insight into Sex Differences in Selenium Metabolism and Risk of Neurodevelopmental Disease. J Neurosci.

[7] Li JL, Li HX, Gao XJ, Zhang JL, Li S, Xu SW, Tang ZX (2012). Priority in selenium homeostasis involves regulation of SepSecS transcription in the chicken brain. PLoS One.

[8] Lu Z, Marks E, Chen J, Moline J, Barrows L, Raisbeck M, Volitakis I, Cherny RA, Chopra V, Bush AI, Hersch S, Fox JH (2014). Altered selenium status in Huntington's disease: neuroprotection by selenite in the N171–82Q mouse model. Neurobiol Dis.

[9] Li JL, Li HX, Li S, Gao XJ, Xu SW, Tang ZX (2012). Effects of Selenoprotein W gene expression by selenium involves regulation of mRNA stability in chicken embryos neurons. Biometals.

[10] Burk RF, Hill KE, Motley AK, Winfrey VP, Kurokawa S, Mitchell SL, Zhang W (2014). Selenoprotein P and apolipoprotein E receptor-2 interact at the blood-brain barrier and also within the brain to maintain an essential selenium pool that protects against neurodegeneration. FASEB J.

[11] Mehta SL, Kumari S, Mendelev N, Li PA (2012). Selenium preserves mitochondrial function, stimulates mitochondrial biogenesis, and reduces infarct volume after focal cerebral ischemia. BMC Neurosci.

[12] Berr C, Arnaud J, Akbaraly TN (2012). Selenium and cognitive impairment: a brief-review based on results from the EVA study. Biofactors.

[13] Byrns CN, Pitts MW, Gilman CA, Hashimoto AC, Berry MJ (2014). Mice lacking selenoprotein P and selenocysteine lyase exhibit severe neurological dysfunction, neurodegeneration, and audiogenic seizures. J Biol Chem.

[14] Tobe R, Carlson BA, Huh JH, Castro NP, Xu XM, Tsuji PA, Lee SG, Bang J, Na JW, Kong YY, Beaglehole D, Southon E, Seifried H (2017). Biochemical characterization of the selenoproteome in Gallus gallus via bioinformatics analysis: structure–function relationships and interactions of binding molecules. Metallomics.

[15] Nakayama A, Hill KE, Austin LM, Motley AK, Burk RF (2007). All regions of mouse brain are dependent on selenoprotein P for maintenance of selenium. J Nutr.

[16] Schweizer U, Schomburg L, Savaskan NE (2004). The neurobiology of selenium: lessons from transgenic mice. J Nutr.

[17] Schweizer U, Bräuer AU, Köhrle J, Nitsch R, Savaskan NE (2004). Selenium and brain function: a poorly recognized liaison. Brain Res Brain Res Rev.

[18] Valentine WM, Abel TW, Hill KE, Austin LM, Burk RF (2008). Neurodegeneration in mice resulting from loss of functional selenoprotein P or its receptor apolipoprotein E receptor 2. J Neuropathol Exp Neurol.

[19] Caito SW, Milatovic D, Hill KE, Aschner M, Burk RF, Valentine WM (2011). Progression of neurodegeneration and morphologic changes in the brains of juvenile mice with selenoprotein P deleted. Brain Res.

[20] Jiang XQ, Cao CY, Li ZY, Li W, Zhang C, Lin J, Li XN, Li JL (2017). Delineating hierarchy of selenotranscriptome expression and their response to selenium status in chicken central nervous system. J Inorg Biochem.

[21] Turanov AA, Xu XM, Carlson BA, Yoo MH, Gladyshev VN, Hatfield DL (2011). Biosynthesis of selenocysteine, the 21st amino acid in the genetic code, and a novel pathway for cysteine biosynthesis. Adv Nutr.

[22] Xu XM, Carlson BA, Irons R, Mix H, Zhong N, Gladyshev VN, Hatfield DL (2007). Selenophosphate synthetase 2 is essential for selenoprotein biosynthesis. Biochem J.

[23] Lobanov AV, Hatfield DL, Gladyshev VN (2008). Selenoproteinless animals: selenophosphate synthetase SPS1 functions in a pathway unrelated to selenocysteine biosynthesis. Protein Sci.

[24] Tobe R, Carlson BA, Huh JH, Castro NP, Xu XM, Tsuji PA, Lee SG, Bang J, Na JW, Kong YY, Beaglehole D, Southon E, Seifried H (2016). Selenophosphate synthetase 1 is an essential protein with roles in regulation of redox homoeostasis in mammals. Biochem J.

[25] Tamura T, Yamamoto S, Takahata M, Sakaguchi H, Tanaka H, Stadtman TC, Inagaki K (2004). Selenophosphate synthetase genes from lung adenocarcinoma cells: Sps1 for recycling L-selenocysteine and Sps2 for selenite assimilation. Proc Natl Acad Sci USA.

[26] Sunde RA, Sunde GR, Sunde CM, Sunde ML, Evenson JK (2015). Cloning, Sequencing, and Expression of Selenoprotein Transcripts in the Turkey (Meleagris gallopavo). PLoS One.

[27] Gueroui M, Kechrid Z (2016). Evaluation of Some Biochemical Parameters and Brain Oxidative Stress in Experimental Rats Exposed Chronically to Silver Nitrate and the Protective Role of Vitamin E and Selenium. Toxicol Res.

[28] Ammar EM, Couri D (1981). Acute toxicity of sodium selenite and selenomethionine in mice after ICV or IV administration. Neurotoxicology.

[29] Rasekh HR, Davis MD, Cooke LW, Mazzio EA, Reams RR, Soliman KF (1997). The effect of selenium on the central dopaminergic system: a microdialysis study. Life Sci.

[30] Wang M, Fu H, Xiao Y, Ai B, Wei Q, Wang S, Liu T, Ye L, Hu Q (2013). Effects of low-level organic selenium on lead-induced alterations in neural cell adhesion molecules. Brain Res.

[31] Liu MC, Xu Y, Chen YM, Li J, Zhao F, Zheng G, Jing JF, Ke T, Chen JY, Luo WJ (2013). The effect of sodium selenite on lead induced cognitive dysfunction. Neurotoxicology.

[32] Labunskyy VM, Hatfield DL, Gladyshev VN (2014). Selenoproteins: molecular pathways and physiological roles. Physiol Rev.

[33] Pavlidou E, Salpietro V, Phadke R, Hargreaves IP, Batten L, McElreavy K, Pitt M, Mankad K, Wilson C, Cutrupi MC, Ruggieri M, McCormick D, Saggar A, Kinali M (2016). Pontocerebellar hypoplasia type 2D and optic nerve atrophy further expand the spectrum associated with selenoprotein biosynthesis deficiency. Eur J Paediatr Neurol.

[34] Chapple C.E, Guigo R (2008). Relaxation of selective constraints causes independent selenoprotein extinction in insect genomes. PLoS One.

[35] Alsina B, Serras F, Baguñá J, Corominas M (1998). patufet, the gene encoding the Drosophila melanogaster homologue of selenophosphate synthetase, is involved in imaginal disc morphogenesis. Mol Gen Genet.

[36] Morey M, Corominas M, Serras F (2003). DIAP1 suppresses ROS-induced apoptosis caused by impairment of the selD/sps1 homolog in Drosophila. J Cell Sci.

[37] Alsina B, Corominas M, Berry MJ, Baguñà J, Serras F (1999). Disruption of selenoprotein biosynthesis affects cell proliferation in the imaginal discs and brain of Drosophila melanogaster. J Cell Sci.

[38] Serras F, Morey M, Alsina B, Baguñà J, Corominas M (2001). The Drosophila selenophosphate synthetase (selD) gene is required for development and cell proliferation. Biofactors.

[39] Shim MS, Kim JY, Jung HK, Lee KH, Xu XM, Carlson BA, Kim KW, Kim IY, Hatfield DL, Lee BJ (2009). Elevation of glutamine level by selenophosphate synthetase 1 knockdown induces megamitochondrial formation in Drosophila cells. J Biol Chem.

[40] Lee KH, Shim MS, Kim JY, Jung HK, Lee E, Carlson BA, Xu XM, Park JM, Hatfield DL, Park T, Lee BJ (2011). Drosophila selenophosphate synthetase 1 regulates vitamin B6 metabolism: prediction and confirmation. BMC Genomics.

[41] Pedersen KS, Kristensen TN, Loeschcke V, Petersen BO, Duus JØ, Nielsen NC, Malmendal A (2008). Metabolomic signatures of inbreeding at benign and stressful temperatures in Drosophila melanogaster. Genetics.

[42] Morey M, Serras F, Corominas M (2003). Halving the selenophosphate synthetase gene dose confers hypersensitivity to oxidative stress in Drosophila melanogaster. FEBS Lett.

[43] Neumuller RA, Richter C, Fischer A, Novatchkova M, Neumüller KG, Knoblich JA (2011). Genome-wide analysis of self-renewal in Drosophila neural stem cells by transgenic RNAi. Cell Stem Cell.

[44] Fortier ME, Audet I, Giguère A, Laforest JP, Bilodeau JF, Quesnel H, Matte JJ (2012). Effect of dietary organic and inorganic selenium on antioxidant status, embryo development, and reproductive performance in hyperovulatory first-parity gilts. J Anim Sci.

[45] Xiao X, Yuan D, Wang YX, Zhan XA (2016). The Protective Effects of Different Sources of Maternal Selenium on Oxidative Stressed Chick Embryo Liver. Biol Trace Elem Res.

[46] Han YH, Zhang ZW, Shao C, Li S, Xu SW, Wang XL (2012). The expression of chicken selenoprotein W, selenocysteine-synthase (SecS), and selenophosphate synthetase-1 (SPS-1) in CHO-K1 cells. Biol Trace Elem Res.

[47] Savaskan NE, Ufer C, Kühn H, Borchert A (2007). Molecular biology of glutathione peroxidase 4: from genomic structure to developmental expression and neural function. Biol Chem.

[48] Renko K, Werner M, Renner-Müller I, Cooper TG, Yeung CH, Hollenbach B, Scharpf M, Köhrle J, Schomburg L, Schweizer U (2008). Hepatic selenoprotein P (SePP) expression restores selenium transport and prevents infertility and motor-incoordination in Sepp-knockout mice. Biochem J.

[49] Diamond AM (2015). The subcellular location of selenoproteins and the impact on their function. Nutrients.

[50] Bermingham EN, Hesketh JE, Sinclair BR, Koolaard JP, Roy NC (2014). Selenium-enriched foods are more effective at increasing glutathione peroxidase (GPx) activity compared with selenomethionine: a meta-analysis. Nutrients.

[51] Tetteh A.Y, Sun KH, Hung CY, Kittur FS, Ibeanu GC, Williams D, Xie J (2014). Transcriptional Response of Selenopolypeptide Genes and Selenocysteine Biosynthesis Machinery Genes in Escherichia coli during Selenite Reduction. Int J Microbiol.

[52] Selenius M, Rundlöf AK, Olm E, Fernandes AP, Björnstedt M (2010). Selenium and the selenoprotein thioredoxin reductase in the prevention, treatment and diagnostics of cancer. Antioxid Redox Signal.

[53] Mangoura D, Dawson G (1993). Opioid peptides activate phospholipase D and protein kinase C-epsilon in chicken embryo neuron cultures. Proc Natl Acad Sci USA.

[54] Hasunuma R, Ogawa T, Kawanishi Y (1982). Fluorometric determination of selenium in nanogram amounts in biological materials using 2,3-diaminonaphthalene. Anal Biochem.

[55] Liu J, Rozovsky S (2015). Membrane-bound selenoproteins. Antioxid Redox Signal.

[56] Blanquicett C, Kang BY, Ritzenthaler JD, Jones DP, Hart CM (2010). Oxidative stress modulates PPAR gamma in vascular endothelial cells. Free Radic Biol Med.

[57] Li JL, Li HX, Li S, Jiang ZH, Xu SW, Tang ZX (2011). Selenoprotein W gene expression in the gastrointestinal tract of chicken is affected by dietary selenium. Biometals.

