# Supplementary material for: Selenophosphate synthetase 1 (SPS1) is required for the development and selenium homeostasis of central nervous system in chicken (Gallus gallus)
Source: Oncotarget. 2017 Mar 16;8(22):35919–32. doi: 10.18632/oncotarget.16283 (PMC5482627; doi:10.18632/oncotarget.16283)
Supplement: Supplementary file 1 [file oncotarget-08-35919-s001.pdf]

Selenophosphate synthetase 1 (SPS1) is required for the development and selenium homeostasis of central nervous system in chicken (*Gallus gallus*)

Supplementary Materials

Supplementary Table 1: Effect of dietary Se level on the Se concentration of CNS tissues (µg/g, wet wt of tissue)

Parameter	0d	15d			25d			35d		
	C-Se	L-Se	C-Se	H-Se	L-Se	C-Se	H-Se	L-Se	C-Se	H-Se
**Cerebral cortex**	0.205 ± 0.004	0.094 ± 0.009^{a}	0.114 ± 0.021^{a}	0.161 ± 0.004^{b}	0.103 ± 0.011^{a}	0.134 ± 0.012^{b}	0.159 ± 0.023c	0.084 ± 0.019^{a}	0.111 ± 0.016^{b}	0.127 ± 0.012b
**Cerebral nuclei**	0.353 ± 0.013	0.066 ± 0.017^{a}	0.113 ± 0.028^{b}	0.213 ± 0.032c	0.067 ± 0.006^{a}	0.138 ± 0.017^{b}	0.177 ± 0.035c	0.095 ± 0.01^{a}	0.115 ± 0.005^{ab}	0.138 ± 0.026b
**Thalamus**	0.19 ± 0.003	0.076 ± 0.017^{a}	0.133 ± 0.006^{b}	0.152 ± 0.012^{b}	0.096 ± 0.002^{a}	0.129 ± 0.01^{b}	0.167 ± 0.019c	0.081 ± 0.013^{a}	0.116 ± 0.004^{b}	0.141 ± 0.026c
**Cerebellum**	0.203 ± 0.002	0.065 ± 0.01^{a}	0.164 ± 0.01^{b}	0.181 ± 0.027^{b}	0.101 ± 0.005^{a}	0.169 ± 0.027^{b}	0.168 ± 0.002^{b}	0.092 ± 0.001^{a}	0.114 ± 0.002^{b}	0.146 ± 0.007c
**Brain stem**	0.181 ± 0.006	0.136 ± 0.018^{a}	0.224 ± 0.001^{b}	0.246 ± 0.008^{b}	0.091 ± 0.015^{a}	0.109 ± 0.005^{a}	0.152 ± 0.024^{b}	0.09 ± 0.002^{a}	0.111 ± 0.01^{b}	0.145 ± 0.04c
**Medulla oblongata**	0.246 ± 0.027	0.174 ± 0.005^{a}	0.209 ± 0.013^{b}	0.206 ± 0.003^{b}	0.095 ± 0.008^{a}	0.149 ± 0.002^{b}	0.169 ± 0.002c	0.092 ± 0.012^{a}	0.131 ± 0.015^{b}	0.171 ± 0.015c
**Marrow**	0.159 ± 0.016	0.048 ± 0.008^{a}	0.162 ± 0.012^{b}	0.214 ± 0.046c	0.051 ± 0.012^{a}	0.152 ± 0.004^{b}	0.184 ± 0.021c	0.07 ± 0.005^{a}	0.135 ± 0.019^{b}	0.161 ± 0.016b
**Sciatic nerve**	0.243 ± 0.002	0.022 ± 0.003^{a}	0.097 ± 0.002^{b}	0.346 ± 0.005c	0.055 ± 0.01^{a}	0.097 ± 0.013^{b}	0.158 ± 0.014c	0.043 ± 0.006^{a}	0.092 ± 0.021^{b}	0.109 ± 0.001c

Data represent mean 6 standard deviation (n = 3/group). Within the groups treated with various levels of Se (L-Se group was fed with low Se diet which contained 0.033 mg/kg Se; C-Se group was fed with the diet containing 0.15 mg/kg Se; H-Se group was fed with the diet containing 1.5 mg/kg Se), data sharing a common letter (a or b or c) are not significantly different ($P < 0.05$).

Supplementary Table 2: Pearson correlation coefficient between tissues Se level and the SPS1 mRNA expression of CNS tissuesw

Parameter	SPS1 mRNA expression level	
	Chicken with low and high Se	Chicken with supernutritional Se
**Cerebral cortex**	0.292	–0.034
**Cerebral nuclei**	–0.219	0.456
**Thalamus**	0.229	0.275
**Cerebellum**	–0.401*	0.666**
**Brain stem**	–0.207	0.578*
**Medulla oblongata**	–0.292	--
**Marrow**	0.669**	--
**Sciatic nerve**	0.319	--

Chicken with low and high Se was fed with Se granulated diet which contained 0.033 mg/kg, 0.15 mg/kg and 1.5 mg/kg Se. Chicken with supernutritional Se was fed with either the commercial granulated diet which contained 0.15 mg/kg Se (Control) or the Se-supplemented diet containing 1.0 (Se-S- I), 2.0 (Se-S- II), 3.0 (Se-S- III) or 5.0 (Se-S- IV) mg/kg sodium selenite. Note: *$P < 0.05$; **$P < 0.01$.w
